# Nutri-Score in the European Food Retail Supply: A Potential Incentive for Food Reformulation?

**DOI:** 10.3390/nu16234184

**Published:** 2024-12-03

**Authors:** Elly Steenbergen, Joline W. J. Beulens, Elisabeth H. M. Temme

**Affiliations:** 1 Centre for Prevention, Lifestyle and Health, National Institute for Public Health and the Environment, 3721 MA Bilthoven, The Netherlands; liesbeth.temme@rivm.nl; 2 Department of Epidemiology & Data Science, Amsterdam University Medical Centre, Location Vrije Universiteit, 1105 AZ Amsterdam, The Netherlands; j.beulens@amsterdamumc.nl; 3 Amsterdam Public Health Research Institute, 1105 AZ Amsterdam, The Netherlands

**Keywords:** food reformulation, front-of-pack nutritional label, Nutri-Score, food composition, food retail supply, Europe

## Abstract

Background: To improve consumers’ diet, policy measures such as food reformulation strategies and front-of-pack nutritional labels (FOPNLs) are implemented, aiming to guide consumers’ food choice and to stimulate an improvement in food composition by manufacturers. The FOPNL Nutri-Score has been implemented in several European countries. Changes in food compositions in relation to the Nutri-Score over time have been limitedly studied. This study evaluates food compositions in Europe over time, and if changes in compositions of the food supply could have potentially resulted in changes in Nutri-Score classifications of foods. Materials and Methods: Food composition data were available from EUREMO, from which bread products, breakfast cereals, hot sauces, and processed potato products from Austria, Belgium, Finland, Italy, and the United Kingdom from 2019 to 2021 were selected (*n* = 2260). Of these countries, only Belgium had implemented the Nutri-Score in 2019. Distributions of food compositions and Nutri-Score classifications were calculated and changes in median salt, sugar, and saturated fatty acids content were plotted by food group, country and year. Distribution of the final sum of Nutri-Score points was plotted by nutrient, food group, country and year. Results: Overall, more favourable Nutri-Score classifications (i.e., towards Nutri-Score classification A) were observed in most of the selected food groups and countries over the years, due to the influence of specific nutrients such as salt in breakfast cereals (lower median of 0.1–0.4 g/100 g) and processed potato products (lower median of 0.1–1.2 g/100 g); and sugar in processed potato products (lower median of 0.1–1.9 g/100 g) and bread products (lower median of 0.7–2.2 g/100 g). For nutrient contents in other food groups, no consistent changes were observed. Conclusions: Changes in the compositions of the food supply resulted in favourable changes in Nutri-Score classifications, suggesting a potential for food reformulation. Monitoring after the actual implementation of Nutri-Score is recommended.

## 1. Introduction

Foods high in energy, salt, sugar and saturated fat contribute to unhealthy diets. Unhealthy diets in turn contribute to overweight and obesity, which are major preventable risk factors for non-communicable diseases such as type II diabetes and cardiovascular diseases. To improve consumers’ diet, the World Health Organization (WHO) has set guidelines to characterize healthy diets [1,2,3,4,5] as well as recommendations on food policy strategies to be implemented by governments to improve consumers’ dietary intake [6].

One food policy strategy is to encourage food manufacturers to reformulate foods to improve their nutritional composition. In Europe, national governments have implemented different reformulation strategies, often targeting the salt, sugar and saturated fat contents of foods by voluntary agreements with the industry to reduce contents or by setting upper limits by law [7,8,9]. Monitoring reformulation efforts is conducted by studying the compositions of branded foods. Several European studies and Joint Actions, such as Best practice in Reformulation, Marketing and Public Procurement (Best-ReMaP) [10] and Joint Action on Nutrition and Physical Activity (JANPA) [11], have collected branded food data in order to map and monitor the nutritional quality of foods to promote reformulation. The European Reformulation Monitoring study (EUREMO) was a study in which the salt, sugar and fat contents of branded foods were included and monitored in 16 European countries in order to provide tools for monitoring and assessing reformulation efforts [12].

Besides encouraging food reformulation, the WHO also recommends the implementation of a front-of-pack nutritional label (FOPNL) [13]. FOPNLs aim to guide consumers in making informed food choices. Meanwhile, FOPNLs also aim to stimulate food manufacturers to improve food compositions in order to apply a more favourable label on the packaging [14]. Several FOPNLs have been developed and implemented in Europe, such as the Heart symbol, the multiple traffic light system, and Nutri-Score. Nutri-Score was initially developed in France in 2017, as the official national FOPNL [15]. It has since been adopted in several other European countries, such as Belgium, Spain, Germany and the Netherlands [16]. An international governance structure, consisting of a steering committee, scientific committee and technical committee, assesses and updates Nutri-Score based on scientific evidence while incorporating and supporting requests and knowledge from stakeholders [17].

Nutri-Score is based on the Food Standards Agency Nutrient Profiling system in the United Kingdom which allocates points based on the food’s nutritional composition. Positive points are allocated to favourable components (i.e., protein, fibre and fruit, vegetables and legumes contents), whereas negative points are allocated to less favourable components (i.e., energy, salt, sugar and saturated fat contents). Three categories of Nutri-Score’s underlying algorithm (i.e., general foods, beverages, and fats, oils, nuts and seeds) calculate the Nutri-Score classification of a food, depending on the type of food, in a final sum of Nutri-Score points and its corresponding letter and colour, ranging from dark green A to dark orange E [18].

Although its implementation and alignment with food-based dietary guidelines has been debated, Nutri-Score was found to be successful in guiding consumers in their food choice in several European countries [19,20,21,22,23]. However, the ability of FOPNLs to encourage food manufacturers to improve foods has not yet been studied in depth [24] or only by scenario analyses. A previous modelling study in France found improved nutrient intakes by the population when substituting food purchases with more favourable alternatives based on Nutri-Score [25]. Previous studies from the Netherlands using data from before the actual implementation of Nutri-Score have shown that changes in the composition of foods result in a change towards a more favourable Nutri-Score classification. In a theoretical study, fixed changes in either the sodium, sugar or saturated fatty acids content resulted in potentially improved Nutri-Score classifications of foods for all foods groups except for legumes, using the original Nutri-Score algorithm from 2017 [22]. A recent Dutch study has shown actual changes in the nutrient compositions of branded foods between 2018 and 2020 while using the updated (2022) Nutri-Score algorithm, which resulted in changes towards more favourable Nutri-Score classifications for breakfast cereals, meat preserves, and sweets and sweet goods [26]. These results from the Netherlands indicate that Nutri-Score may be effective as an incentive for food manufacturers to stimulate food reformulation when implemented. However, only the Dutch retail supply was included.

These results need to be confirmed by data from other European countries to assess whether Nutri-Score could be a potential incentive for food reformulation for the European food retail supply. Therefore, the present study evaluates food compositions in several European countries over time and if changes in food composition would have resulted in changes in Nutri-Score classifications of foods.

## 2. Materials and Methods

### 2.1. Data Preparation

EUREMO data were used for the analyses, extraction date 7 February 2024. This dataset includes food compositions of food items categorized by food group and subgroup from several European countries (i.e., Austria, Belgium, Bulgaria, Denmark, Estonia, Finland, France, Greece, Hungary, Italy, Lithuania, Malta, Portugal, Romania, Slovenia, and the United Kingdom) from the years 2011 to 2022. Food composition data were extracted from images of food labels using applications of extraction software. Food groups covered 50% or more of the market share by country [12]. More detailed information on the background of EUREMO data and its data collection can be found elsewhere [12].

**Figure 1:**
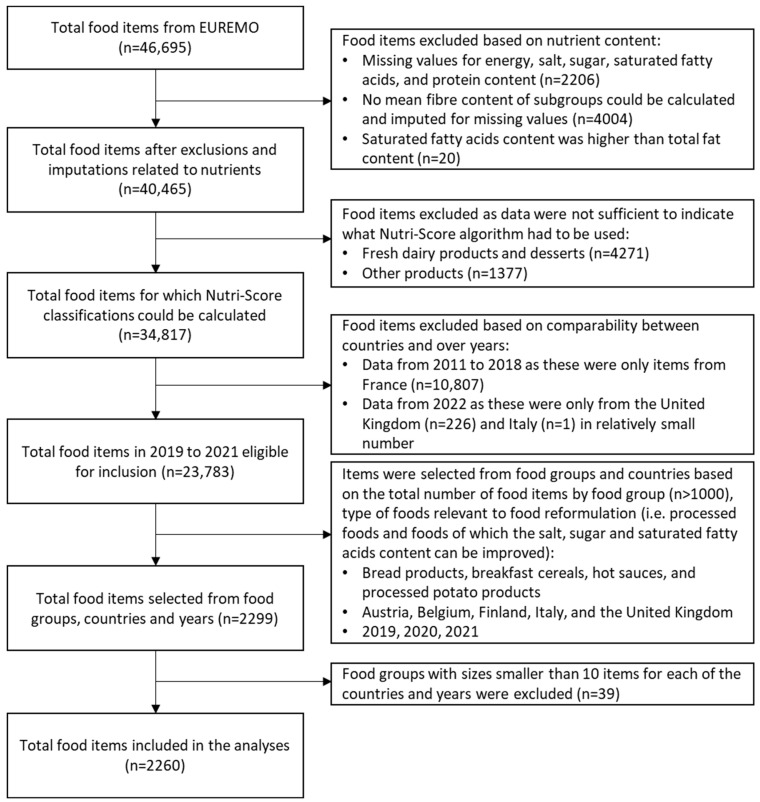
Flow chart of the selection of food items included in the analyses.

Figure 1 shows a flowchart of the selection procedure of foods included in the analyses. From the EUREMO dataset, data on Nutri-Score classifications were not available. Therefore, Nutri-Score classifications for foods had to be calculated. Food items were excluded when contents lacked the nutrients needed for the calculation of the Nutri-Score classification (i.e., energy, salt, saturated fatty acids, sugar, protein), except for fibre and the fruit, vegetables and legumes content (not mandatory on the back-of-pack) [27]. Additionally, the food groups fresh dairy products and desserts, and other products (heterogenous food group) were excluded as data were not sufficient to indicate and differentiate the type of food (e.g., drink or a solid food) as to whether the Nutri-Score classifications should be calculated using either the general, beverages or fats, oils, nuts and seeds algorithm (Nutri-Score guidelines state different calculation rules for solid foods, drinks or dairy products considered as added fats). Data from the years 2011 to 2018 only include foods sold in France and are of different food groups in each of these years. As these foods could not be compared over years, foods from France were excluded from the analyses (Table A1 in Appendix A). In 2022, only data from the UK and Italy were available and in a relatively small number, and were therefore excluded from the analyses. The majority of the data were from the years 2019 to 2021, in which a selection of food groups and countries was made for the analyses based on the total number of food items by food group (*n* > 1000), type of foods relevant to food reformulation (i.e., processed foods and foods of which the salt, sugar and saturated fatty acids content can be improved), and availability of food items in the years 2019 to 2021 by country (Table A2 in Appendix A). The food groups bread products, breakfast cereals, hot sauces and processed potato products from the countries Austria, Belgium, Finland, Italy and the United Kingdom were selected. Of the selected countries, Belgium is the only country that has implemented Nutri-Score (in 2019). Of the selected food groups, food group sizes smaller than 10 items in a specific country and year were excluded from the analyses.

For the analyses, assumptions have been made for the remaining missing values. For fibre content, the mean content of the subgroup was imputed for missing values. The fruit, vegetables and legumes content was assumed by subgroup for the calculation of the Nutri-Score (Table A3 in Appendix A).

### 2.2. Data Analyses

As there was no unique identifier available for the food items in the dataset to establish whether the same foods were available over the years, changes in compositions of these same foods within food groups over time could not be evaluated. Therefore, the number and type of items within subgroups of food groups were compared over the years (Table A3 in Appendix A). For this reason, descriptive results were provided to compare changes over time. The analyses involved the calculation of the distribution of nutrient contents by food group, country and year. For the salt, sugar and saturated fatty acids content (in g/100 g), changes in median contents over 2019–2021 were plotted by food group and country as these three nutrients are often targeted in reformulation strategies. Nutri-Score classifications were calculated for foods according to Nutri-Score guidelines [28] and their distributions were calculated by food group, country and year. The distributions of the final sum of Nutri-Score points were calculated and plotted by the salt, sugar and saturated fatty acids contents by food group, country and year.

All statistical analyses were performed in R software, version 4.4.1.

## 3. Results

An overview of the number of food items by selected food group, country and year is shown in Table 1. For Italy, data on bread products from only 2020 were included in the analyses. A total of 2260 foods were included in the analyses with group sizes ranging from 10 to 183 foods by food group, country and year (Table 1). Over the years, the proportion of subgroups within food groups changed (Table A3 in Appendix A). Pre-packaged breads within bread products increased over time in Austria (from 41/74 to 88/100) and the United Kingdom (from 17/25 to 46/56), whereas plain rusks decreased in Finland (from 101/183 to 24/179). The proportion of high-fibre cereals within breakfast cereals in Finland decreased over the years (from 22/67 to 4/22). Within hot sauces, numbers of tomato coulis and similar increased in Austria (from 0/31 to 10/38) and Italy (from 3/24 to 19/55) over time, whereas pesto sauces, sauces with tomatoes and cheese, sweet and sour sauces and other sauces from around the world decreased to zero in Finland in 2020. The proportion of French fries for the deep fryer within processed potato products increased in all selected countries over the years (Table A3 in Appendix A).

**Figure 2:**
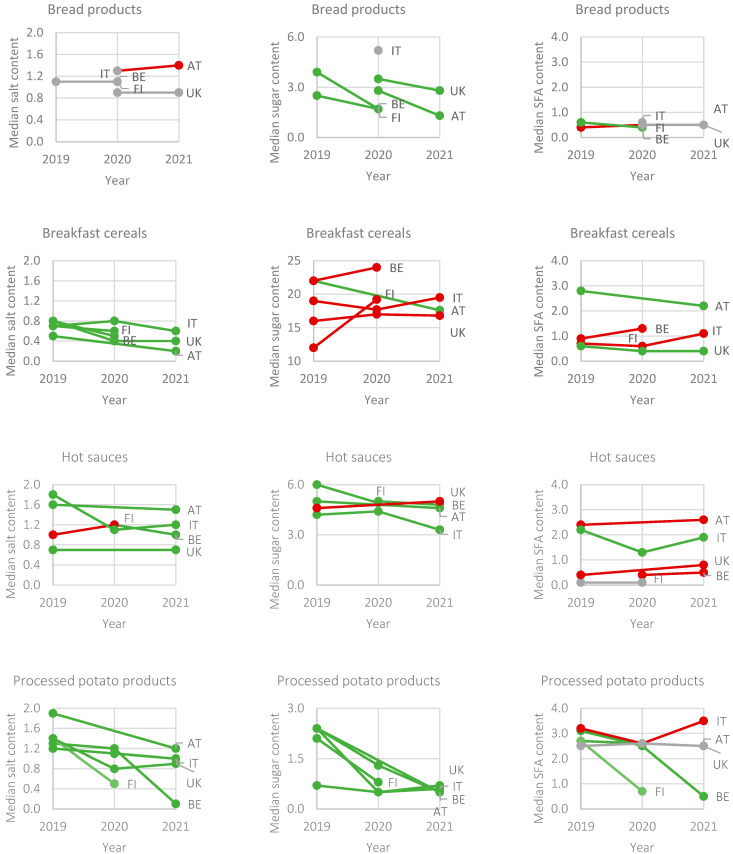
Changes in median salt, sugar and saturated fatty acids (SFAs) contents (g/100 g) by food group and country over years 2019 to 2021. Green lines indicate decreased nutrient contents, red lines indicate increased nutrient contents and grey lines indicate unchanged nutrient contents. AT = Austria, BE = Belgium, FI = Finland, IT = Italy, UK = United Kingdom. See Table A4 in Appendix A for the distributions of nutrient contents by food group, country and year.

Table A4 in Appendix A shows the distributions of the nutrient contents of each of the selected food groups, countries and years. Differences in the median salt, sugar and saturated fatty acids contents of food groups by country between 2019 and 2021 are shown in Figure 2. Lower median contents were observed for each of the three nutrients as well as unchanged and higher median nutrient contents in the most recent year. Over time, bread products had an unchanged median salt content in all countries, except for Austria. Bread products contained less sugar in all countries (median difference of 0.7–2.2 g/100 g). In addition, bread products in Finland contained less saturated fatty acids over the years. For breakfast cereals, the median salt content was lower with 0.1–0.4 g/100 g in all countries over the years, whereas the sugar content was only lower in Austria (Table A4 in Appendix A and Figure 2). In Austria, breakfast cereals improved in all three selected nutrients as the three median nutrient contents were lower in the most recent year. Hot sauces in all countries, except for Finland, had a lower median salt content (median difference of 0.1–0.6 g/100 g) by 2021. Except for the United Kingdom, the median sugar content of hot sauces was also lower (0.2–1.1 g/100 g) over the years. Italy was the only country with a lower median saturated fatty acids content of hot sauces over time (Table A4 in Appendix A and Figure 2). Processed potato products have improved in median salt (median difference of 0.1–1.2 g/100 g) and sugar (median difference of 0.1–1.9 g/100 g) content in all countries over the years. Additionally, processed potato products from Belgium and Finland had lower median contents for all three nutrients. Italy was the only country in which one of the median nutrient contents was higher (i.e., median saturated fatty acids content increased by 0.3 g/100 g) (Table A4 in Appendix A and Figure 2).

**Figure 3:**
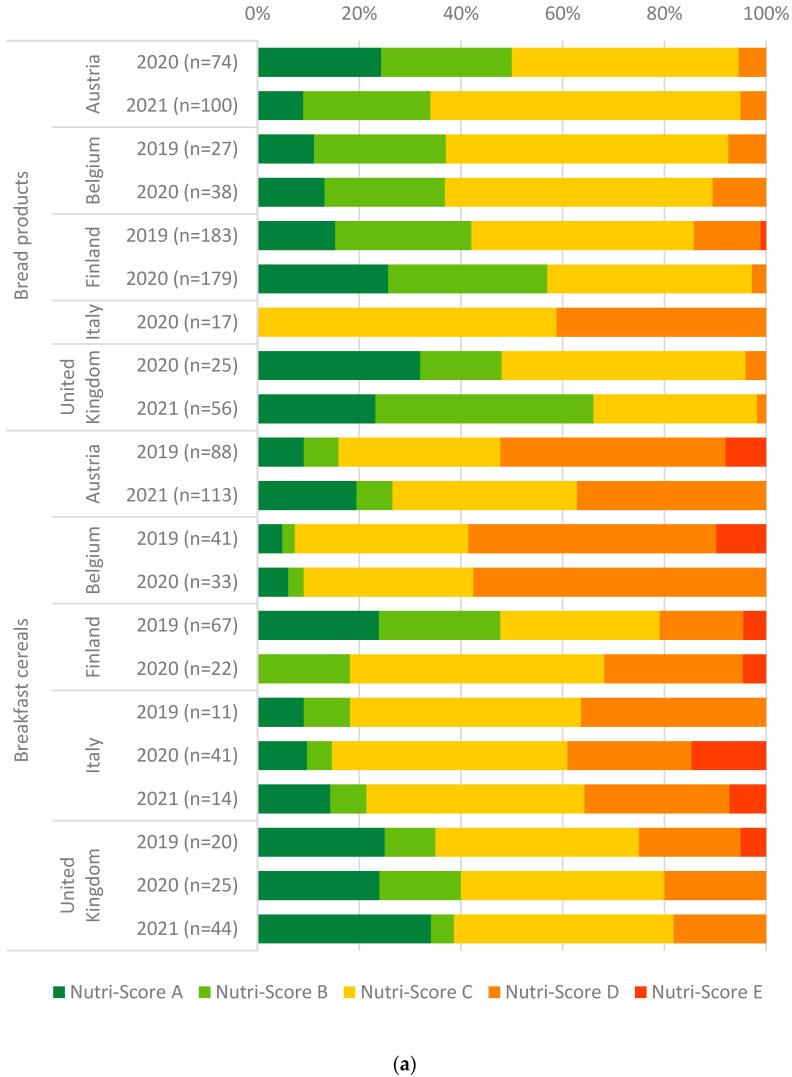

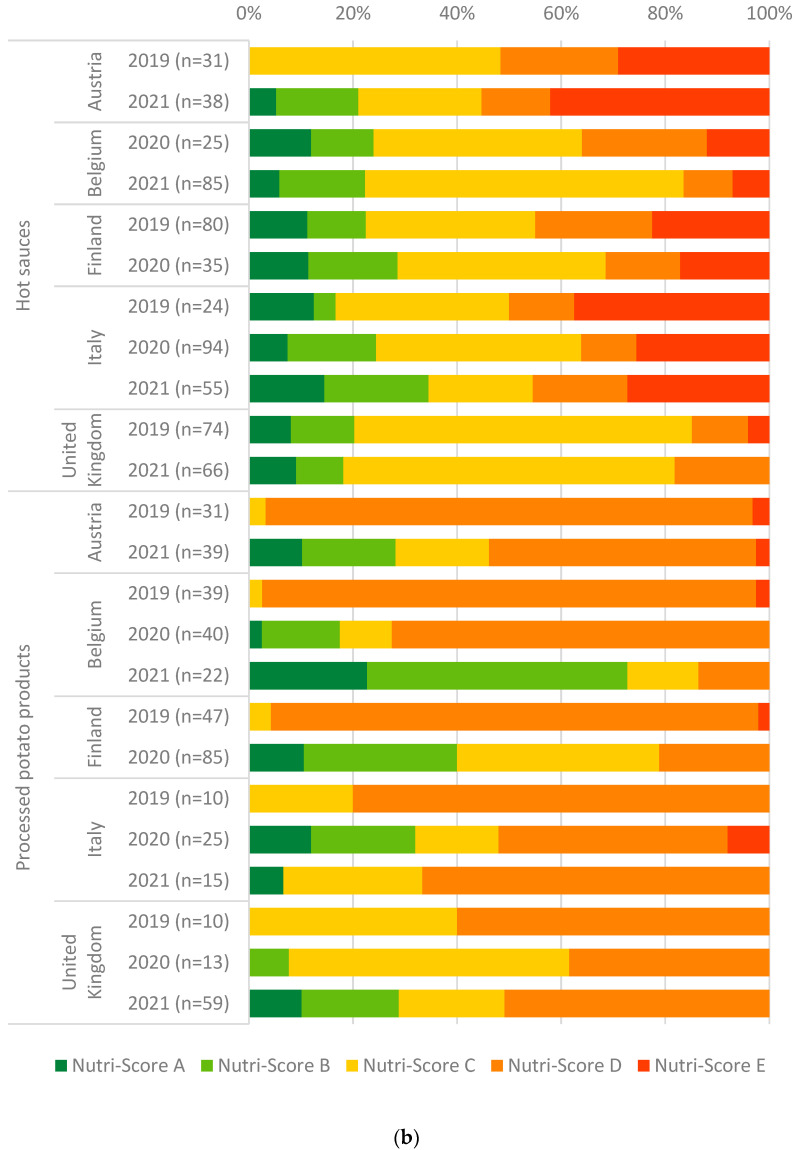
(**a**). Distribution of Nutri-Score classifications calculated for bread products and breakfast cereals by country and year. (**b**). Distribution of Nutri-Score classifications calculated for hot sauces and processed potato products by country and year.

Figure 3a,b and Table A5 in Appendix A show the distributions of calculated Nutri-Score classifications of food groups by country and year. The distributions show a shift towards a more favourable Nutri-Score classification (i.e., towards Nutri-Score classification A) in most of the selected food groups and countries over the years. In Belgium and Finland, bread products shifted towards Nutri-Score classification A through the years (2 and 10 percentage points increase in Nutri-Score classification A, respectively), whereas in Austria and the United Kingdom Nutri-Score classifications unfavourably shifted towards C (16 percentage points increase) and B (9 percentage points increase), respectively. For breakfast cereals, Nutri-Score classifications improved towards A in Austria, Belgium, Italy and the United Kingdom over the years (10, 1, 5 and 9 percentage points increase for Nutri-Score classification A). Breakfast cereals in Finland changed unfavourably towards Nutri-Score classification C and D (19 and 11 percentage points increase). Nutri-Score classifications of hot sauces shifted towards A, especially in Austria (sum of 21 percentage points increase for Nutri-Score classifications A and B), Finland (6 percentage points increase for Nutri-Score classification B), Italy (sum of 18 percentage points increase for Nutri-Score classifications A and B) and the United Kingdom (1 percentage point increase for Nutri-Score classification A). Processed potato products improved towards Nutri-Score classifications A over the years in all countries (sum ranging from 7 to 73 percentage points increase for Nutri-Score classifications A and B). Distributions of Nutri-Score classifications ranged over three to five classifications, except for bread products in Italy in 2020 and processed potato products in Italy and the United Kingdom in 2019. For these latter, Nutri-Score classifications range over two classifications (i.e., C and D).

**Figure 4:**
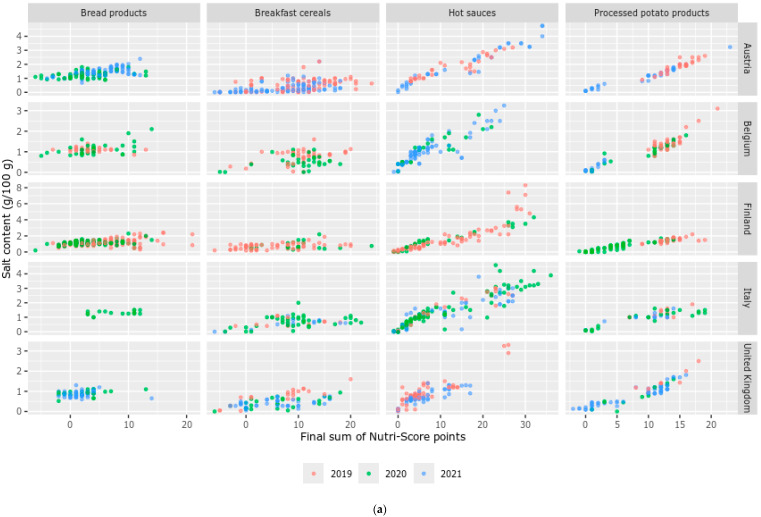

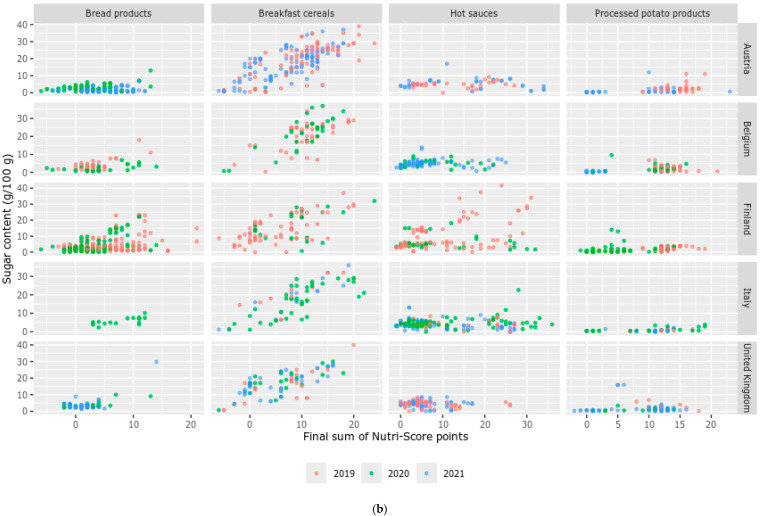

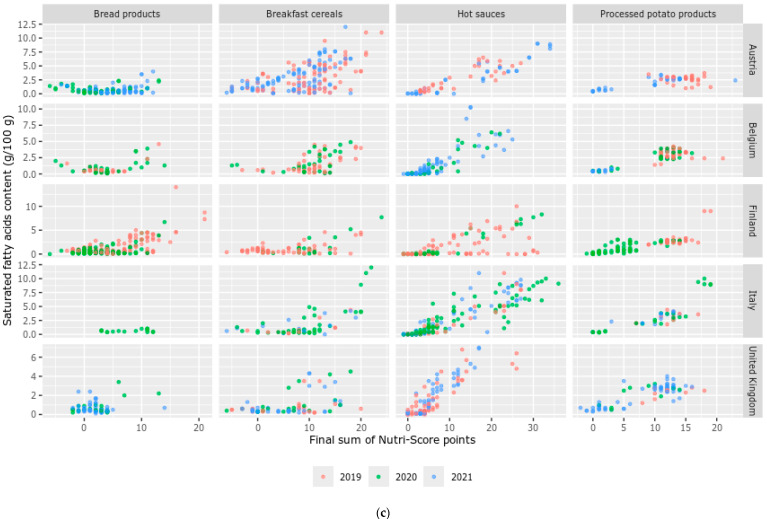
(**a**). Salt content (g/100 g) plotted by the final sum of Nutri-Score points by food group and country from 2019 to 2021. (**b**). Sugar content (g/100 g) plotted by the final sum of Nutri-Score points by food group and country from 2019 to 2021. (**c**) Saturated fatty acids content (g/100 g) plotted by the final sum of Nutri-Score points by food group and country from 2019 to 2021.

Figure 4a–c show the final sum of Nutri-Score points plotted by the salt, sugar and saturated fatty acids contents (respectively) of foods by food group, country and year. These figures show the influence of the specific nutrient alone on the final sum of Nutri-Score points. The influence of the salt content on the final sum of Nutri-Score points of foods is most visible for hot sauces and processed potato products as foods with lower salt content have a lower final sum of Nutri-Score points (i.e., towards Nutri-Score classification A) and foods with higher salt content have a higher final sum of points (Figure 4a). Foods from the most recent year were also found to be more on the left bottom side of the plots than foods from other years of some food groups in countries, showing that foods from more recent years are generally lower in salt content as shown in Figure 3. This can be observed for breakfast cereals in Austria and the United Kingdom, hot sauces in Austria, and processed potato products in Austria, Belgium, Finland and the United Kingdom (Figure 4a).

The influence of the sugar content on the final sum of Nutri-Score points was most visible for bread products and breakfast cereals (Figure 4b), whereas the influence of the saturated fatty acids content of foods was visible in all food groups (Figure 4c). Foods from the most recent year were visibly lower in sugar content and the final sum of Nutri-Score points for breakfast cereals in Austria, hot sauces in Finland and processed potato products in Austria (Figure 3 and Figure 4b). The saturated fatty acids content was lower in the most recent year, especially for breakfast cereals in Austria and processed potato products in Finland (Figure 3 and Figure 4c).

## 4. Discussion

The present study aimed to assess whether Nutri-Score could be a potential incentive to food manufacturers for food reformulation in the European food retail supply, by evaluating food compositions in European countries over time and if changes in compositions of the food retail supply could have resulted in changes in Nutri-Score classifications. Overall, most changes in the median salt, sugar and saturated fatty acids content and Nutri-Score classifications of foods were favourable as observed in the selected food groups and countries from 2019 to 2021. Some unfavourable changes were observed as well, such as the higher sugar content in breakfast cereals in all countries except for Austria. Depending on the food group, specific nutrients influenced the Nutri-Score classification as lower contents resulted in a more favourable Nutri-Score classification (e.g., salt in breakfast cereals and processed potato products) and vice versa.

The results in the present study show that changes in the food supply as well as its food composition have taken place from 2019 to 2021 in several European countries and food groups. These findings are in line with what was previously found in the Netherlands [26], confirming the indications that Nutri-Score classifications could be improved by favourably changing the nutrient composition of foods. As for the other way around, Nutri-Score may act as a disincentive to food manufacturers to worsen the composition of foods.

The changes in food composition may reflect food reformulation by food manufacturers as each of the countries in the present study have implemented reformulation strategies targeting either the salt, sugar or saturated fat content of foods before or during the years 2019 to 2021 [7,8]. One may define food reformulation as the change in food composition of the same food item over time, but food reformulation is also defined as the change in the composition of the food retail supply. This does not necessarily mean the change in food composition of an individual food item, but new food items could also have been introduced that have more favourable compositions [29]. In the present study, it was not possible to distinguish food reformulation as to whether it resulted from the improvement in existing foods or by the introduction of new foods, as a unique identifier of foods in the dataset, such as the European Article Number, was not available. However, the number of food items by subgroup differed for some food groups in several countries over the years. Changes in the composition of the food group may therefore be due to a change in the proportion of subgroups within a subgroup, and not necessarily due to the reformulation of foods. In the present study, food reformulation reflects both the change in the composition of existing food items as well as the change in the composition of the food retail supply due to introductions to or eliminations of food items from the market. This was also found in a previous study from the Netherlands, in which changes in nutrient contents were found to be due to the improvement in existing foods as well as due to the introduction of new food items within the food groups [26]. In Belgium, before Nutri-Score was officially implemented, breakfast cereals had already improved in mean salt (−20%), sugar (−5.2%) and saturated fatty acids (−4%) content. These improvements were found in individual foods that were available in both 2017 and 2018 [30]. The present study showed comparable improvements in the salt content of breakfast cereals in Belgium from 2019 to 2020. However, the sugar and saturated fatty acids content increased during these years (20.5 to 22.3 g and 0.9 to 1.3 g, respectively). Reformulation of breakfast cereals occurred to existing foods and prior to the actual implementation of Nutri-Score in Belgium from 2017 to 2018 [30].

Nutri-Score has currently been implemented in several European countries, but not in all of the countries or during the years that were studied in the present study. Austria and Italy have not officially implemented, or announced the intention to implement Nutri-Score, whereas since 2000 Finland has implemented the Heart symbol [31] and the United Kingdom has had its multiple traffic light system since 2013 [32]. In Belgium, where Nutri-Score has been implemented since 2019 [33], the implementation of Nutri-Score has impacted efforts by food manufacturers to improve food compositions of breakfast cereals as observed in a previous study [30]. Studies on the effects of FOPNLs on food reformulation by food manufacturers are still limited. A study suggested improved food compositions of store-brand cakes by household purchases in Great Britain after the implementation of the multiple traffic light system [24]. Other FOPNLs in countries may have also had an impact on the observed food compositions since foods in Europe are regularly exported to other countries. Additionally, national reformulation strategies during these years may have resulted in the improvements that were found in the food compositions. As Nutri-Score was only implemented in Belgium during the years studied, the present study does not show the efforts that food manufacturers might put into food reformulation when Nutri-Score is actually implemented. Besides, Nutri-Score classifications were calculated for all foods available of the selected food groups. In practice, Nutri-Score may not be applied to all foods as it is still a voluntary food policy measure. Food manufacturers may choose not to apply Nutri-Score on their foods, for example when they only manufacture foods that will receive unfavourable Nutri-Score classifications. A mandatory approach on the use of FOPNLs may have more impact as suggested before [34]. Several national food reformulation strategies are also voluntary [7] and may therefore not incentivize all food manufacturers to improve their foods.

Although Belgium was the only country to have implemented Nutri-Score during the years and of the countries studied, the present study shows that nutrient compositions and calculated Nutri-Score classifications have improved for the selected food groups, countries and years. Moreover, changes in the contents of specific nutrients seemed to be reflected by Nutri-Score classifications and their change over the years. This suggests that Nutri-Score could potentially incentivize food manufacturers to improve food compositions. These efforts may show even more when monitoring food compositions in countries which have already implemented the Nutri-Score. Therefore, it would be highly recommended to monitor the nutrient compositions and Nutri-Score classifications of foods after the actual implementation of Nutri-Score. Monitoring compositions of the food retail supply and Nutri-Score classifications of foods after its implementation will validate whether Nutri-Score is able to incentivize food manufacturers to improve foods, especially when looking at foods that are available in several years.

The present study used EUREMO data, which consist of food composition data of several food groups from several countries and years. As composition data were not (sufficiently) available for all food groups, countries and years, a selection was made for the analyses in this study. In addition, the dataset did not provide sufficient information to establish and determine the Nutri-Score algorithm to be used to calculate classifications for fresh dairy products and desserts according to Nutri-Score guidelines. For the analyses regarding Nutri-Score, the fruit, vegetables and legumes content of foods was not available and therefore assumed, as well as the missing fibre content of foods for which the mean fibre content was imputed for missing contents by subgroup. Calculated Nutri-Score classifications may therefore deviate from actual values. Additionally, in this dataset, foods were already categorized into food groups, which combined a minimum of 50% of the market share. Therefore, not the whole food retail supply was included in the dataset, even though the weighted selection aimed to establish a sample representative of actual purchases and consumption [12]. The dataset did also not provide sufficient information to identify and establish whether foods were available in multiple years, although the type of foods were known within food groups over the years. Yet, comparisons of identical foods over years were not possible. As the data collection of foods within food groups was based on market share, the proportion of types of foods available within food groups in countries and years differed for some of the food groups. This limitation may have affected the results regarding the changes (both favourable and unfavourable) that were observed for food compositions and calculated Nutri-Score classifications, although most of these changes represented the actual food retail supply. Furthermore, some changes were rather large for the relatively short period of time. This could be due to changes in market shares of food items and in combination with smaller food group sizes. These changes should also be interpreted cautiously. The EUREMO dataset has its strengths as it includes data on branded food items, rather than generic food items, and from multiple European countries over multiple years. Using food composition data of foods from different brands, food groups and different countries provides valuable insights into the composition of the European food retail supply.

## 5. Conclusions

The present study showed favourable changes in the median salt, sugar, and saturated fatty acids content in several of the selected food groups (bread products, breakfast cereals, hot sauces and processed potato products) and countries (Austria, Belgium, Finland, Italy and the UK) from 2019 to 2021. With these changes, in the case of Nutri-Score classifications being applied on all foods under study, Nutri-Score classifications would have shifted towards more favourable classifications. These findings suggest that Nutri-Score could be a potential incentive for food manufacturers to improve food compositions. To establish whether and to what extent food manufacturers are incentivized by Nutri-Score to improve foods, monitoring of nutrient composition and Nutri-Score classifications of foods over years, especially after Nutri-Score is implemented, is recommended.

## Figures and Tables

**Table 1:** Overview of the number of food items by selected food group, country and year.

Food Group	Country	N in 2019	N in 2020	N in 2021
Bread products	Austria	0	74	100
	Belgium	27	38	0
	Finland	183	179	0
	Italy	9	17	9
	United Kingdom	9	25	56
Breakfast cereals	Austria	88	9	113
	Belgium	41	33	1
	Finland	67	22	0
	Italy	11	41	14
	United Kingdom	20	25	44
Hot sauces	Austria	31	2	38
	Belgium	0	25	85
	Finland	80	35	0
	Italy	24	94	55
	United Kingdom	74	0	66
Processed potato products	Austria	31	0	39
	Belgium	39	40	22
	Finland	47	85	0
	Italy	10	25	15
	United Kingdom	10	13	59

N = number of food items. Grey cells indicate the food groups of a specific country and year in which the group size is smaller than 10 items. These were not included in the analyses of this study.

## Data Availability

The Food and Beverages Labels Explorer (FABLE) is an information system developed by the Joint Research Centre (JRC) of the European Commission. Collection of data hosted in FABLE, and used for this research, was supported with funding from the European Union’s Health Programme (2014–2020) through the EUREMO project. The data are available on request via https://food-labels-explorer.jrc.ec.europa.eu/en, accessed on 25 November 2024.

## Appendix A

**Table A1:** Number of food items by year and country with data available on energy, salt, sugar, saturated fatty acids and protein content and either available or assumed fibre content from the EUREMO database.

	Austria	Belgium	Bulgaria	Denmark	Estonia	Finland	France	Greece	Hungary	Italy	Lithuania	Malta	Portugal	Romania	Slovenia	United Kingdom	Total
2011							612										612
2012							489										489
2013							1029										1029
2015							4177										4177
2016							1136										1136
2017							3015										3015
2018							349										349
2019	923	197				1581				118						510	3329
2020	267	606	1231	1751	1534	1802		2527	1682	1018	1323	603	1738	134	2696	446	19,358
2021	1706	1023				694			20	425	361	718	277	510	5	1005	6744
2022										1						226	227
Total	2896	1826	1231	1751	1534	4077	10,807	2527	1702	1562	1684	1321	2015	644	2701	2187	40,465

Grey rows indicate the years for which data were selected for the analyses in the study.

**Table A2:** Number of food items by country and food groups with data available on energy, salt, sugar, saturated fatty acids and protein content and either available or assumed fibre content from the EUREMO database from 2019 to 2021.

Country	Bread Products	Breakfast Cereals	Cakes and Biscuits	Canned Fruits	Cheeses	Chocolate Products	Cold Sauces	Confectionery	Crackers	Delicatessen Meats and Similar	Fresh Delicatessen Products	Frozen Snacking Products	Fruit Juices and Nectars	Hot Sauces	Ice Creams and Sorbets	Processed Potato Products	Ready-to-Eat Canned Meals	Ready-to-Eat Fresh Meals	Soft Drinks	Soups and Broths	Total
2019																					
Austria		88	21	15		136	63		50	118				31	13	31			112		678
Belgium	27	41		22					20							39			48		197
Finland	183	67	35	15		198	96	52	81		13			80	37	47		10	255	6	1175
Italy	9	11		1		29	2	6	9		1			24		10			5		107
United Kingdom	9	20				74	28		13	38				74	46	10	16	1	35		364
Total	228	227	56	53		437	189	58	173	156	14			209	96	137	16	11	455	6	2521
2020																					
Austria	74	9	20		115				10		3	34		2							267
Belgium	38	33	85	13	7	91	32		18	99	8	16		25	50	40			27		582
Bulgaria	44	56	179		28	345	23	51	52	98	11			8	147	43		4	23	25	1137
Denmark	111	146	166	10	58	276	96	140	10	269		53		93	120	27			29		1604
Estonia	58	120	135	10	91	241	33		25		83	43			229	51	132	5		27	1283
Finland	179	22	269	15	47	23	38	83	45	299	146	19		35	59	85		151	48	136	1699
Greece	113	114	266	10	281	358	113		205	206	34	130	17	101	63	90			101		2202
Hungary	44	139	190	9	26	234	67		66	195	45	55		37	148	62	29	40	56		1442
Italy	17	41	115	4	5	75	9	27	27	59	25	51		94	181	25	19	6	71		851
Lithuania	95	63	101	8	39	188	81		71	76	66	28	71	46	107	69	27	6	71		1213
Malta	31	141	35		6	99	11	33	60	11		11		15		40	15		9		517
Portugal	42	102	266	4	68	271	52	58	52	30	44	71		51	109	79	29	17	85		1430
Romania	6	26	7			8	2			26	3				2	20	4				104
Slovenia	102	91	419	13	271	232	98		181	20	8	46		102	353	87	97	27	127	95	2369
United Kingdom	25	25	92		28				7		29	12				13	73		28	66	398
Total	979	1128	2345	96	1070	2441	655	392	829	1388	505	569	88	609	1568	731	425	256	675	349	17,098
2021																					
Austria	100	113	104	27	274	225	49		24	318	9	33		38	18	39	81		76		1528
Belgium		1	154	5	12	252	64		6	209	7	12		85	93	22		4	14		940
Finland										163		116			369			16			664
Hungary	4	4	9											2		1					20
Italy	9	14	68	3	12	40	5	5	8	31	1	17		55	30	15	15	1	11		340
Lithuania	31	40	10			83	16		3	4				27	44	21	9		29		317
Malta	18	145	45		62	19	31		20	90	39	39		12	59	35	15		31		660
Portugal	9	3	74	3	7	53			24	7	1	14		1	38	7	2	13	5		261
Romania	41	65	160	3	2	12	14		21	30	3	5		1		42	8	5	51		463
Slovenia						1											3				4
United Kingdom	56	44	63		70	43	27		30	114	37	32		66	32	59	33	6	93	55	860
Total	268	429	687	41	439	728	206	5	136	966	97	268		287	683	241	166	45	310	55	6057
*Total*	*1475*	*1784*	*3088*	*190*	*1509*	*3606*	*1050*	*455*	*1138*	*2510*	*616*	*837*	*88*	*1105*	*2347*	*1109*	*607*	*312*	*1440*	*410*	*25,676*

Grey rows indicate the countries and food groups from which data were selected for the analyses in the study.

**Table A3:** Overview of total number of food items in food groups and subgroups, and number of foods with fibre content available, mean fibre content (g/100 g) and assumed fruit, vegetables and legumes content (%) by subgroup, country and year.

Food Group	2019	2020	2021	Subgroup Name	2019			2020			2021			Assumed FVL Content
	N_total_	N_total_	N_total_		N_total_	N_fibre_	Mean_fibre_	N_total_	N_fibre_	Mean_fibre_	N_total_	N_fibre_	Mean	
Austria														
Bread products	0	74	100	Plain white sandwich breads/hamburger/hot dog buns				14	14	2.8	5	1	3.0	≤40
				Pre-baked breads				9	6	3.8	4	1	2.5	≤40
				Pre-packaged breads				41	36	8.7	88	47	6.6	≤40
				Wholemeal_cereal_grains sandwich breads/hamburger/hot dog buns				8	8	5.6	3	1	3.4	≤40
Breakfast cereals	88	9	113	Cereal flakes with chocolate_nuts	2	2	4.2	1	1	2.5	1	1	5.8	≤40
				Cereal flakes with fruit	1	1	5.4							≤40
				Cereals without added sugar	5	5	5.4				7	7	7.8	≤40
				Chocolate and caramel cereals				1	1	6.7	1	1	6.7	≤40
				Chocolate-flavoured cereals	8	8	6.2	1	1	6	9	7	7.0	≤40
				Crunchy chocolate muesli	20	20	7.8				14	12	7.7	≤40
				Crunchy fruit muesli	10	10	9.1				10	10	8.7	≤40
				Crunchy muesli with nuts_seeds	8	8	9.1				15	14	7.1	≤40
				Filled cereals	7	7	4.2				1	0		≤40
				High-fibre cereals	3	3	7.1	2	2	8.3	3	3	7.7	≤40
				Honey/caramel cereals	9	9	6.0	1	1	5	6	6	9.1	≤40
				Sweet cereal flakes	3	3	4.6	3	3	3.3	8	6	3.2	≤40
				Traditional muesli flakes	12	12	9.2				39	34	6.9	≤40
Hot sauces	31	2	38	Bolognese sauces and similar	3	3	1.3				3	3	0.5	≤40
				Other hot sauces							3	3	0.4	≤40
				Pesto sauces	15	15	3.0				17	17	1.9	≤40
				Tomato coulis and similar				2	1	1.2	10	8	1.3	≤60
				Sauces with tomatoes and cheese	3	3	1.8							≤40
				Tomato sauces	8	8	1.6				5	3	1.4	≤60
				Tomato_olive sauces	2	2	2.6							≤60
Processed potato products	31	0	39	Classic and wavy crisps	5	3	4.6				23	21	4.4	≤40
				French fries (chips) for deep fryer							12	11	2.9	≤40
				Low-fat crisps	5	1	0.5				4	4	5.0	≤40
Belgium														≤40
Bread products	27	38	0	Plain white sandwich breads/hamburger/hot dog buns	6	6	5.7	2	1	3.2				≤40
				Pre-baked breads	2	2	2.5	7	5	3.8				≤40
				Pre-packaged breads	17	13	6.3	26	25	5.2				≤40
				Wholemeal_cereal_grains sandwich breads/hamburger/hot dog buns	2	2	7.2	3	3	4.4				≤40
Breakfast cereals	41	33	1	Cereal flakes with chocolate_nuts	3	3	4.9	3	3	5.3				≤40
				Cereal flakes with fruit	1	1	5.3	1	1	5.9				≤40
				Cereals without added sugar	1	1	5.0	2	2	9.6				≤40
				Chocolate and caramel cereals	2	2	6.0	1	1	5.3				≤40
				Chocolate-flavoured cereals	14	14	6.5	9	9	5.4				≤40
				Filled cereals	5	5	3.7	5	5	5.1				≤40
				High-fibre cereals	4	4	8.1	1	1	6.3				≤40
				High-fibre fruit cereals	1	1	9.0	3	3	9.3				≤40
				Honey/caramel cereals	8	8	5.3	5	5	5.4				≤40
				Sweet cereal flakes	2	2	3.0	3	2	4.1	1	1	3.8	≤40
Hot sauces	0	25	85	Bolognese sauces and similar				3	2	2.3	9	5	1.4	≤40
				Other hot sauces				1	1	0.5	2	2	0.3	≤40
				Pesto sauces				5	2	3.1	9	8	2.6	≤40
				Sauces with tomatoes and cheese				2	1	1.0	7	5	1.3	≤40
				Sweet and sour sauces				1	0		2	2	0.8	≤40
				Tomato coulis and similar				8	6	1.1	18	18	1.7	≤60
				Tomato sauces				4	3	1.2	36	29	1.6	≤60
				Tomato_olive sauces				2	1	1.5	2	1	2.6	≤60
Processed potato products	39	40	22	Classic and wavy crisps	37	28	4.4	30	19	4.2	3	2	4.2	≤40
				French fries (chips) for deep fryer				10	9	2.8	19	19	2.5	≤40
				Low-fat crisps	2	2	4.6							≤40
Finland														≤40
Bread products	183	179	0	Other_sandwich breads/hamburger/hot dog buns				2	2	6.2				≤40
				Plain rusks	101	94	12.5	24	23	14.1				≤40
				Plain white sandwich breads/hamburger/hot dog buns	4	4	3.7	1	1	3.0				≤40
				Pre-baked breads	1	1	2.4	3	3	2.7				≤40
				Pre-packaged breads	53	51	5.9	141	138	7.2				≤40
				Wholemeal_cereal_grains sandwich breads/hamburger/hot dog buns	24	24	6.4	8	8	6.3				≤40
Breakfast cereals	67	22	0	Cereal flakes with chocolate_nuts	1	1	5.0							≤40
				Cereal flakes with fruit	2	2	4.5							≤40
				Cereals without added sugar	4	4	9.7							≤40
				Chocolate and caramel cereals	1	1	6.5							≤40
				Chocolate-flavoured cereals	13	13	7.4	7	7	7.2				≤40
				Filled cereals	3	3	3.1	3	3	3.7				≤40
				High-fibre cereals	22	22	9.1	4	4	7.1				≤40
				High-fibre fruit cereals	9	9	9.7	2	2	9.6				≤40
				Honey/caramel cereals	6	6	5.3	3	2	8.8				≤40
				Sweet cereal flakes	6	6	3.2	3	2	3.4				≤40
Hot sauces	80	35	0	Bolognese sauces and similar	1	0	1	0						≤40
				Other hot sauces	1	1	0.5	10	5	5.3				≤40
				Other sauces from around the world	17	1	0.3							≤40
				Pesto sauces	19	3	3.1							≤40
				Sauces with tomatoes and cheese	7	3	1.6							≤40
				Sweet and sour sauces	8	4	0.7							≤40
				Tomato coulis and similar	20	1	0.5	13	5	2.4				≤60
				Tomato sauces	8	1	1.1	10	7	1.4				≤60
				Tomato_olive sauces				2	1	1.8				≤60
Processed potato products	47	85	0	Classic and wavy crisps	47	11	4.2	19	9	4.9				≤40
				French fries (chips) for deep fryer				24	17	2.4				≤40
				Low-fat crisps	1	0		1	0					≤40
				Other processed potato products				12	10	1.9				≤40
				Potato croquettes and balls				1	1	3.0				≤40
				Ready-to-eat mashed potatoes				8	5	1.8				≤40
				Röstis				2	2	2.3				≤40
				Sautéed potatoes				19	10	2.7				≤40
Italy														≤40
Bread products	9	17	9	Plain white sandwich breads/hamburger/hot dog buns	5	5	3.0	11	11	3.0	7	7	2.5	≤40
				Pre-baked breads	1	1	2.5	1	0					≤40
				Wholemeal_cereal_grains sandwich breads/hamburger/hot dog buns	3	3	6.6	6	6	4.6	2	2	3.6	≤40
Breakfast cereals	11	41	14	Cereal flakes with chocolate_nuts	2	2	5.2							≤40
				Cereal flakes with fruit				2	2	6.0	2	2	10.4	≤40
				Cereals without added sugar				2	2	7.5	2	2	8.4	≤40
				Chocolate-flavoured cereals	1	1	6.1	10	10	5.8	5	5	6.0	≤40
				Filled cereals	1	1	5.3	4	4	3.7	1	1	2.1	≤40
				High-fibre cereals				1	1	8.7				≤40
				Honey/caramel cereals	3	3	16.8	10	10	11.2	1	1	33.0	≤40
				Sweet cereal flakes	4	4	6.6	12	12	5.8	3	3	3.5	≤40
Hot sauces	24	94	55	Bolognese sauces and similar	5	5	1.1	19	12	1.5	7	4	0.8	≤40
				Other hot sauces	2	2	1.0	3	2	0.7	5	3	1.0	≤40
				Pesto sauces	8	5	1.4	28	15	2.8	16	16	2.0	≤40
				Sauces with tomatoes and cheese				1	1	1.5				≤40
				Tomato coulis and similar	3	3	1.4	20	15	1.7	19	19	1.2	≤60
				Tomato sauces	5	5	1.3	24	15	2.6	8	8	1.7	≤60
				Tomato_olive sauces	1	1	1.3							≤60
Processed potato products	10	25	15	Classic and wavy crisps	10	10	4.2	16	16	4.6	14	14	5.0	≤40
				French fries (chips) for deep fryer				8	7	2.2	1	1	2.5	≤40
				Low-fat crisps				1	1	2.5				≤40
United Kingdom														≤40
Bread products	9	25	56	Other_sandwich breads/hamburger/hot dog buns				2	2	2.8				≤40
				Other breads							1	1	4.1	≤40
				Plain white sandwich breads/hamburger/hot dog buns	1	1	2.3	2	2	2.6	5	5	2.6	≤40
				Pre-baked breads				4	4	4.0	2	2	3.7	≤40
				Pre-packaged breads	8	8	4.5	17	17	4.5	46	46	4.5	≤40
				Wholemeal_cereal_grains sandwich breads/hamburger/hot dog buns							2	2	6.7	≤40
Breakfast cereals	20	25	44	Cereal flakes with chocolate_nuts				1	1	2.3	2	2	2.4	≤40
				Cereal flakes with fruit	1	1	5.3	1	1	3.5	3	3	3.7	≤40
				Cereals without added sugar	2	2	12.5	1	1	13.0				≤40
				Chocolate-flavoured cereals	1	0		5	5	7.3	3	3	4.2	≤40
				Filled cereals				5	5	6.6	7	7	6.5	≤40
				High-fibre cereals	8	8	12.5	4	4	11.4	10	10	13.1	≤40
				High-fibre fruit cereals	1	1	9.0	1	1	8.4	4	4	8.8	≤40
				Honey/caramel cereals	5	5	8.9	3	3	9.5	7	7	4.3	≤40
				Sweet cereal flakes	3	3	3.5	4	4	3.1	8	8	2.8	≤40
Hot sauces	74	0	66	Bolognese sauces and similar	22	22	1.3				10	10	1.2	≤40
				Other hot sauces	5	5	0.5				7	7	0.5	≤40
				Pesto sauces	11	11	2.3				14	14	2.7	≤40
				Sauces with tomatoes and cheese	4	4	1.1				6	6	1.1	≤40
				Tomato coulis and similar	7	6	1.1				6	6	1.1	≤60
				Tomato sauces	25	24	1.9				23	23	1.4	≤60
				Tomato_olive sauces	1	1	0.7							≤60
Processed potato products	10	13	59	Classic and wavy crisps	8	5	4.2	9	9	4.2	31	31	4.2	≤40
				French fries (chips) for deep fryer				3	3	3.0	23	23	3.6	≤40
				Low-fat crisps	2	2	6.3	1	1	3.7	5	5	3.1	≤40

N_total_ = total number of food items. N_fibre_ = number of food items with fibre content available. Mean_fibre_ = mean fibre content (g/100 g). FVL content = fruit, vegetables and legumes content (%). Grey cells indicate the food groups of a specific country and year in which the group size is smaller than 10 items. These were not included in the analyses of this study.

**Table A4:** (**a**). Distribution of the energy content (kJ/100 g), salt content (g/100 g) and sugar content (g/100 g) of selected food groups by country and year. (**b**). Distribution of the saturated fatty acids, fibre and protein content (g/100 g) of selected food groups by country and year.

(a)																					
				Energy Content (kJ/100 g)						Salt Content (g/100 g)						Sugar Content (g/100 g)					
Food Group	Country	Year	N	Mean	P5	P25	P50	P75	P95	Mean	P5	P25	P50	P75	P95	Mean	P5	P25	P50	P75	P95
Bread products	Austria	2020	74	1006	803	931	1043	1100	1178	1.2	0.9	1.1	1.3	1.3	1.6	3.1	0.9	1.8	2.8	3.9	5.7
		2021	100	1095	880	981	1085	1160	1357	1.5	1.0	1.3	1.4	1.6	1.9	1.7	0.4	0.7	1.3	2.4	4.2
	Belgium	2019	27	1117	1011	1057	1108	1140	1254	1.1	1.0	1.0	1.1	1.1	1.2	4.7	1.4	2.7	3.9	5.4	10.1
		2020	38	1175	976	1045	1121	1192	1712	1.1	0.8	0.9	1.1	1.2	1.6	2.2	0.6	1.0	1.7	2.8	5.1
	Finland	2019	183	1365	907	1066	1397	1538	2125	1.2	0.7	1.0	1.1	1.3	1.8	4.0	0.8	1.4	2.5	3.9	15.0
		2020	179	1115	814	994	1067	1174	1514	1.1	0.8	1.0	1.1	1.2	1.4	3.2	0.4	0.9	1.7	3.1	13.9
	Italy	2020	17	1154	1049	1129	1167	1185	1232	1.3	1.0	1.3	1.3	1.4	1.5	5.8	3.5	4.5	5.2	7.5	8.0
	United Kingdom	2020	25	1094	940	987	1058	1171	1315	0.9	0.7	0.8	0.9	1.0	1.1	4.0	2.3	3.0	3.5	4.1	8.4
		2021	56	1089	935	1010	1071	1140	1338	0.9	0.7	0.8	0.9	1.0	1.1	3.7	2.1	2.6	2.8	3.6	7.0
Breakfast cereals	Austria	2019	88	1734	1531	1612	1732	1850	1970	0.5	0.0	0.2	0.5	0.8	1.0	19.4	0.9	14.0	22.0	25.3	33.0
		2021	113	1589	577	1538	1660	1804	1974	0.3	0.0	0.1	0.2	0.5	0.9	16.5	1.0	10.0	17.6	22.0	29.0
	Belgium	2019	41	1662	1560	1604	1633	1681	1892	0.8	0.2	0.7	0.8	1.0	1.1	20.5	7.0	17.0	22.0	25.0	29.0
		2020	33	1661	1564	1608	1623	1710	1876	0.5	0.0	0.4	0.5	0.7	1.0	22.3	3.7	17.7	24.0	27.0	34.8
	Finland	2019	67	1578	1374	1556	1602	1631	1744	0.7	0.2	0.4	0.7	0.9	1.2	14.1	3.4	8.4	12.0	18.5	28.7
		2020	22	1647	1578	1600	1616	1654	1863	0.8	0.4	0.5	0.6	1.0	1.5	17.3	5.8	8.9	19.2	24.7	28.5
	Italy	2019	11	1603	1425	1560	1596	1637	1799	0.7	0.4	0.5	0.7	0.9	1.1	20.3	6.5	15.3	19.0	28.0	32.0
		2020	41	1669	1522	1588	1629	1684	1992	0.7	0.0	0.4	0.8	1.0	1.1	16.8	4.0	8.5	17.7	24.9	28.9
		2021	14	1620	1471	1564	1604	1676	1785	0.6	0.0	0.5	0.6	0.8	1.0	17.5	1.1	11.8	19.5	22.0	31.5
	United Kingdom	2019	20	1568	1484	1529	1578	1603	1653	0.8	0.0	0.6	0.8	1.0	1.2	15.1	0.7	8.0	16.0	20.5	25.8
		2020	25	1582	1416	1514	1571	1639	1760	0.4	0.1	0.2	0.4	0.6	0.7	17.5	4.8	14.0	17.0	23.0	28.6
		2021	44	1589	1407	1520	1601	1636	1799	0.4	0.1	0.3	0.4	0.5	0.7	16.5	6.1	11.8	16.8	21.0	27.3
Hot sauces	Austria	2019	31	950	272	341	881	1571	1800	1.8	0.8	1.0	1.6	2.4	3.1	4.8	1.4	4.2	5.0	5.7	7.6
		2021	38	1026	99	189	1273	1514	2324	2.0	0.2	0.8	1.5	3.2	4.1	5.3	1.0	3.7	4.6	7.0	8.3
	Belgium	2020	25	565	104	177	309	485	1688	1.2	0.4	0.5	1.2	1.6	2.2	4.9	1.8	3.1	5.0	6.1	8.5
		2021	85	436	128	197	298	402	1541	1.0	0.4	0.7	1.0	1.2	2.5	5.0	2.5	4.0	4.8	5.7	7.2
	Finland	2019	80	639	97	229	393	805	1884	1.6	0.1	0.4	1.0	2.1	5.3	10.6	1.9	3.8	6.0	14.0	29.0
		2020	35	734	129	150	240	1578	2534	1.4	0.0	0.6	1.2	1.9	3.6	5.8	1.8	3.5	4.9	5.8	15.0
	Italy	2019	24	896	124	227	455	1481	2458	1.7	0.1	1.1	1.8	2.5	3.0	4.3	0.4	3.3	4.2	5.2	7.8
		2020	94	798	112	206	398	1274	2427	1.4	0.0	0.7	1.1	1.8	3.4	4.6	1.7	3.2	4.4	5.5	7.5
		2021	55	930	92	139	632	1711	2532	1.3	0.0	0.4	1.2	2.0	3.0	3.8	0.9	2.0	3.3	5.0	8.5
	United Kingdom	2019	74	444	129	192	260	430	1481	0.9	0.1	0.7	0.7	0.9	1.4	4.6	1.2	3.8	4.6	5.7	6.6
		2021	66	493	142	170	340	550	1504	0.7	0.1	0.6	0.7	0.8	1.3	4.5	0.5	3.8	5.0	5.6	7.8
Processed potato products	Austria	2019	31	2124	1793	2097	2155	2201	2262	1.8	1.2	1.6	1.9	2.0	2.5	2.8	0.5	1.7	2.4	2.5	8.8
		2021	39	1741	639	849	2106	2269	2304	1.1	0.1	0.5	1.2	1.6	2.0	1.2	0.2	0.5	0.5	1.3	3.0
	Belgium	2019	39	2170	1971	2109	2213	2237	2310	1.4	0.9	1.2	1.3	1.5	2.2	2.2	0.5	0.7	2.4	3.2	4.0
		2020	40	1789	515	1723	2166	2234	2268	1.0	0.1	0.8	1.2	1.3	1.5	1.8	0.0	0.5	1.3	2.3	4.7
		2021	22	826	522	556	589	701	2212	0.4	0.1	0.1	0.1	0.5	1.2	0.6	0.0	0.4	0.5	0.5	1.0
	Finland	2019	47	2225	2077	2199	2230	2273	2325	1.4	1.0	1.3	1.4	1.6	1.8	2.2	0.4	1.5	2.1	3.2	3.8
		2020	85	941	311	500	630	862	2253	0.6	0.0	0.0	0.5	1.1	1.5	1.5	0.2	0.5	0.8	1.8	3.7
	Italy	2019	10	2192	2010	2045	2155	2235	2508	1.3	1.0	1.0	1.2	1.5	1.8	0.8	0.2	0.5	0.7	1.0	1.7
		2020	25	1624	529	606	2080	2132	2261	0.9	0.1	0.2	1.1	1.3	1.5	1.1	0.4	0.5	0.5	1.0	3.3
		2021	15	2067	1552	2036	2212	2242	2321	1.0	0.5	1.0	1.0	1.1	1.5	0.7	0.0	0.2	0.6	0.9	2.0
	United Kingdom	2019	10	2072	1834	2069	2094	2171	2211	1.4	0.9	1.1	1.4	1.4	2.3	2.8	0.3	1.0	2.4	3.8	6.4
		2020	13	1806	652	2032	2046	2138	2208	0.8	0.1	0.5	0.8	1.2	1.5	1.3	0.3	0.5	0.5	1.5	3.3
		2021	59	1592	667	799	2039	2157	2243	0.8	0.1	0.4	0.9	1.2	1.7	1.8	0.2	0.5	0.7	1.7	5.4
(**b**)																					
				Saturated Fatty Acids Content (g/100 g)						Fibre Content (g/100 g)						Protein Content (g/100 g)					
Food Group	Country	Year	N	Mean	P5	P25	P50	P75	P95	Mean	P5	P25	P50	P75	P95	Mean	P5	P25	P50	P75	P95
Bread products	Austria	2020	74	0.7	0.2	0.3	0.5	0.8	1.9	6.5	2.5	3.5	5.9	8.7	12.1	9.4	5.3	7.2	8.5	9.8	19.3
		2021	100	0.7	0.1	0.2	0.5	0.8	1.6	6.2	2.6	4.8	6.6	6.6	14.2	9.7	5.1	7.4	8.6	10.8	19.2
	Belgium	2019	27	0.7	0.2	0.4	0.4	0.6	2.1	5.9	2.8	4.0	6.3	7.2	8.8	8.8	7.0	7.6	8.1	9.1	11.9
		2020	38	0.9	0.1	0.3	0.5	1.2	3.5	4.7	2.5	3.2	4.2	5.4	9.3	10.7	7.6	8.5	10.0	12.0	16.2
	Finland	2019	183	1.2	0.2	0.4	0.6	1.2	4.2	9.6	3.0	4.9	7.7	13.0	20.0	9.8	4.1	7.8	9.4	11.5	17.0
		2020	179	0.6	0.1	0.2	0.4	0.7	1.8	7.9	3.0	4.2	6.8	10.5	17.0	9.0	5.0	7.8	8.9	10.0	13.0
	Italy	2020	17	0.6	0.4	0.5	0.6	0.7	1.0	3.6	2.2	2.8	3.0	4.2	6.0	8.9	8.1	8.5	8.5	9.2	10.3
	United Kingdom	2020	25	0.7	0.2	0.3	0.5	0.9	2.2	4.1	1.9	2.4	3.7	5.3	7.0	9.9	8.7	9.1	10.0	10.2	11.8
		2021	56	0.6	0.2	0.3	0.5	0.7	1.7	4.4	2.0	3.0	4.1	5.5	7.4	9.9	8.3	9.0	9.8	11.0	12.1
Breakfast cereals	Austria	2019	88	3.1	0.2	0.9	2.8	5.1	7.3	7.3	3.2	5.5	6.5	8.0	15.0	9.5	6.2	8.0	9.1	10.0	13.7
		2021	113	2.9	0.2	1.0	2.2	4.3	7.4	7.1	1.3	5.8	7.1	8.9	11.4	9.2	4.0	8.1	9.3	11.0	12.5
	Belgium	2019	41	1.5	0.2	0.6	0.9	2.5	4.2	5.8	3.0	4.6	5.3	7.4	9.0	8.0	6.3	7.0	8.0	8.9	10.0
		2020	33	1.8	0.2	0.4	1.3	3.0	4.3	5.9	2.6	4.6	5.5	6.9	10.2	8.4	6.2	7.0	8.2	9.2	11.4
	Finland	2019	67	1.0	0.2	0.4	0.7	1.2	3.7	7.5	2.3	5.0	7.7	10.0	12.7	9.5	6.4	8.2	9.5	11.0	12.7
		2020	22	1.4	0.1	0.3	0.6	1.3	5.1	6.6	2.6	3.4	6.5	8.9	12.0	8.8	6.6	7.6	8.7	10.0	11.5
	Italy	2019	11	1.2	0.2	0.4	0.7	1.2	3.7	9.0	3.2	5.2	5.8	6.9	25.5	8.7	7.0	7.3	8.0	9.5	12.5
		2020	41	2.0	0.3	0.4	0.6	3.0	8.9	7.1	2.2	4.0	5.3	8.4	15.4	8.7	6.1	7.2	8.3	9.0	14.0
		2021	14	1.5	0.1	0.6	1.1	2.4	3.9	8.1	2.2	3.7	5.8	8.2	21.8	9.5	5.7	7.3	8.4	11.9	15.0
	United Kingdom	2019	20	0.8	0.2	0.3	0.6	0.8	3.5	9.7	3.0	5.3	9.0	12.0	17.5	9.9	6.8	8.5	9.7	12.0	12.1
		2020	25	1.1	0.3	0.4	0.4	0.9	4.1	7.3	2.0	3.2	8.3	9.3	14.6	8.8	6.6	7.4	7.9	11.0	12.0
		2021	44	0.9	0.3	0.3	0.4	0.7	3.3	6.6	1.7	2.5	5.6	8.9	15.6	8.8	6.2	7.5	7.9	9.6	12.0
Hot sauces	Austria	2019	31	2.8	0.4	1.0	2.4	5.0	6.1	2.3	1.2	1.5	2.0	2.7	4.8	3.1	1.4	1.6	3.1	4.3	5.4
		2021	38	3.1	0.0	0.1	2.6	4.7	8.9	1.4	0.4	1.0	1.4	1.7	3.0	3.2	1.0	1.5	3.4	4.4	6.4
	Belgium	2020	25	1.7	0.0	0.1	0.4	3.8	6.0	1.7	0.7	1.1	1.2	2.2	3.7	2.4	1.1	1.2	1.8	3.5	4.9
		2021	85	1.4	0.0	0.1	0.5	1.7	6.1	1.7	0.6	1.3	1.6	2.0	2.8	2.4	1.0	1.3	1.6	3.3	6.5
	Finland	2019	80	1.4	0.0	0.0	0.1	2.2	5.7	1.3	0.3	0.5	0.7	1.9	3.1	2.3	0.4	1.1	1.6	3.4	5.1
		2020	35	1.8	0.0	0.1	0.1	3.9	7.4	2.9	1.2	1.5	2.4	4.9	6.6	2.7	1.1	1.5	1.6	4.1	5.2
	Italy	2019	24	3.2	0.0	0.3	2.2	5.0	8.9	1.3	0.7	1.0	1.4	1.5	1.6	3.6	1.1	1.5	3.7	4.8	7.5
		2020	94	2.6	0.0	0.3	1.3	4.8	8.8	2.2	1.0	1.5	1.8	2.7	4.0	3.8	1.0	1.4	2.2	5.1	10.3
		2021	55	3.2	0.0	0.1	1.9	6.1	9.2	1.4	0.7	0.9	1.1	2.0	2.8	4.5	0.9	1.4	2.5	6.2	14.3
	United Kingdom	2019	74	1.2	0.0	0.1	0.4	1.5	5.0	1.5	0.5	0.9	1.3	1.6	2.7	2.2	1.1	1.3	1.6	2.3	4.9
		2021	66	1.6	0.1	0.1	0.8	3.0	4.9	1.5	0.5	0.7	1.5	1.9	3.1	2.1	1.0	1.4	1.6	2.6	4.8
Processed potato products	Austria	2019	31	2.6	1.2	2.5	2.7	3.0	3.4	4.4	3.6	4.3	4.4	4.5	5.3	5.9	4.7	5.7	5.9	6.1	6.6
		2021	39	2.0	0.5	0.9	2.5	2.7	3.0	4.0	2.5	3.4	4.2	4.5	5.7	4.7	2.5	3.5	4.9	5.5	6.5
	Belgium	2019	39	3.1	2.3	2.7	3.1	3.7	3.9	4.4	3.8	4.0	4.3	4.4	5.8	5.8	3.7	5.7	6.0	6.2	6.4
		2020	40	2.3	0.4	2.0	2.5	3.3	3.9	3.8	2.4	3.9	4.1	4.2	4.8	5.1	2.1	4.8	5.9	6.2	6.6
		2021	22	0.9	0.3	0.4	0.5	0.7	3.4	2.7	2.0	2.2	2.5	3.0	4.1	3.0	2.2	2.3	2.5	2.9	6.0
	Finland	2019	47	2.9	2.2	2.5	2.7	2.8	3.4	4.2	4.2	4.2	4.2	4.2	4.7	6.0	5.0	5.7	6.0	6.4	6.9
		2020	85	1.3	0.1	0.4	0.7	2.5	2.9	2.9	1.1	2.0	2.6	3.0	4.9	3.0	1.3	1.7	2.5	3.0	6.0
	Italy	2019	10	3.1	1.9	2.2	3.2	3.7	4.3	4.2	2.5	2.9	4.1	5.0	6.4	6.7	5.4	6.1	6.8	7.3	8.0
		2020	25	3.3	0.4	0.5	2.6	3.8	9.3	3.7	2.0	2.4	3.8	4.9	5.4	5.4	2.1	3.0	6.2	6.9	7.8
		2021	15	3.0	1.4	2.2	3.5	3.7	4.1	4.8	3.3	4.2	4.5	5.8	6.4	6.2	4.3	5.5	6.2	7.3	8.0
	United Kingdom	2019	10	2.3	1.4	2.2	2.5	2.6	2.8	4.6	3.8	4.2	4.2	4.6	6.3	6.4	5.8	6.0	6.4	6.7	7.2
		2020	13	2.2	0.6	1.9	2.6	2.8	3.1	3.8	2.6	3.7	4.0	4.3	4.4	5.4	2.6	4.8	5.8	6.3	7.1
		2021	59	2.0	0.4	0.7	2.5	2.9	3.5	3.9	2.5	3.3	3.7	4.5	5.7	5.0	2.5	3.1	5.1	6.8	8.0

N = number of food items. P5 = 5th percentile. P25 = 25th percentile. P50 = 50th percentile. P75 = 75th percentile. P95 = 95th percentile.

**Table A5:** Distribution of Nutri-Score classifications (A to E, in %) by food group, country and year, and change within Nutri-Score classifications (in percentage points) by food group and country over the years.

		Distribution of Nutri-Score Classifications (A–E, in %)																		Change Over the Years (in Percentage Points)				
		2019						2020						2021										
Food Group	Country	N	A	B	C	D	E	N	A	B	C	D	E	N	A	B	C	D	E	A	B	C	D	E
Bread products	Austria							74	24	26	45	5		100	9	25	61	5		−15	−1	16	0	0
	Belgium	27	11	26	56	7		38	13	24	53	11								2	−2	−3	3	0
	Finland	183	15	27	44	13	1	179	26	31	40	3								10	5	−3	−10	−1
	Italy							17			59	41												
	United Kingdom							25	32	16	48	4		56	23	43	32	2		−9	27	−16	−2	0
Breakfast cereals	Austria	88	9	7	32	44	8							113	19	7	36	37		10	0	4	−7	−8
	Belgium	41	5	2	34	49	10	33	6	3	33	58								1	1	−1	9	−10
	Finland	67	24	24	31	16	4	22		18	50	27	5							−24	−6	19	11	0
	Italy	11	9	9	45	36		41	10	5	46	24	15	14	14	7	43	29	7	5	−2	−3	−8	7
	United Kingdom	20	25	10	40	20	5	25	24	16	40	20		44	34	5	43	18		9	−5	3	−2	−5
Hot sauces	Austria	31			48	23	29							38	5	16	24	13	42	5	16	−25	−9	13
	Belgium							25	12	12	40	24	12	85	6	16	61	9	7	6	16	61	9	7
	Finland	80	11	11	33	23	23	35	11	17	40	14	17							0	6	8	−8	−5
	Italy	24	13	4	33	13	38	94	7	17	39	11	26	55	15	20	20	18	27	2	16	−13	6	−10
	United Kingdom	74	8	12	65	11	4							66	9	9	64	18		1	−3	−1	7	−4
Processed potato products	Austria	31			3	94	3							39	10	18	18	51	3	10	18	15	−42	−1
	Belgium	39			3	95	3	40	3	15	10	73		22	23	50	14	14		23	50	11	−81	−3
	Finland	47			4	94	2	85	11	29	39	21								11	29	35	−72	−2
	Italy	10			20	80		25	12	20	16	44	8	15	7		27	67		7	0	7	−13	0
	United Kingdom	10			40	60		13		8	54	38		59	10	19	20	51		10	19	−20	−9	0

N = number of food items.
